# Efficacy of *Banha-sasim-tang* on functional dyspepsia classified as excess pattern: study protocol for a randomized controlled trial

**DOI:** 10.1186/s13063-017-2282-z

**Published:** 2017-11-09

**Authors:** Yun Hee Kim, Jun Young Kim, So Young Jung, O-Jin Kwon, Jun-Hwan Lee, Chang-Gue Son

**Affiliations:** 1 Korean Medicine Convergence Research Division, Korea Institute of Oriental Medicine (KIOM), Daejeon, 34054 South Korea; 2 Liver and Immunology Research Center, Daejeon Oriental Hospital of Daejeon University, 176-9 Daeheung-ro, Jung-gu, Daejeon, 34929 South Korea; 3 Korean Medicine Clinical Research Division, Korea Institute of Oriental Medicine (KIOM), Daejeon, 34054 South Korea

**Keywords:** Functional dyspepsia, *Banha-sasim-tang*, Excess pattern, Randomized trial

## Abstract

**Background:**

Functional dyspepsia (FD) refers to the presence of one or more gastrointestinal symptoms including postprandial fullness, epigastric pain, burning, and early satiety without an organic explanation for chronic symptoms. FD causes considerable discomfort in patients and affects their everyday activity and productivity. Because most conventional treatments have limited efficacy, numerous patients seek alternatives such as herbal medicines. In this proposed study, we will conduct a clinical trial of an herbal drug, *Banha-sasim-tang* (BST). Furthermore, participants will be limited to those classified as having an excess pattern by using an instrument of pattern identification for FD to determine the efficacy of BST in a specific subset of patients.

**Methods:**

This randomized, blinded, parallel-group clinical trial of BST versus placebo will consist of 4 weeks of oral administration of BST or placebo and a 4-week follow-up period. The Korean version of the symptom-based questionnaire of the Nepean Dyspepsia Index (NDI-K) will be used as the primary outcome measure. Secondary outcome measures will include the quality-of-life (QoL) evaluation from the NDI-K, the FD-related QoL (FD-QoL) scale, assessment of gastric myoelectrical dysrhythmias (GMA), and a Visual Analog Scale (VAS) analysis.

**Discussion:**

The results of this trial are expected to provide relevant evidence demonstrating that BST can be used as an effective treatment in a specific subset of FD subjects.

**Trial registration:**

KCT 0002013. Registered at Clinical Research Information Service in the Republic of Korea on 18 August 2016.

**Electronic supplementary material:**

The online version of this article (doi:10.1186/s13063-017-2282-z) contains supplementary material, which is available to authorized users.

## Background

Dyspepsia, which is characterized by the presence of one or more of the following symptoms, postprandial fullness, epigastric pain, burning, and early satiety, is thought to originate in the gastroduodenal region of the upper gastrointestinal (GI) tract [1]. Studies using upper GI endoscopy have indicated that less than 10%, less than 1%, and more than 70% of patients with dyspepsia had a peptic ulcer, gastroesophageal cancer, and functional dyspepsia (FD), respectively [2]. The chronic symptoms of FD have no organic explanation, occur at least weekly, and last for at least 6 months [3]. The global prevalence of FD is between 5 and 11%, and 40% of patients visit a physician for medication owing to considerable discomfort and detrimental effects on everyday activities and productivity [4–6]. Unfortunately, conventional medicines have limited efficacy, and up to 50% of patients with FD seek alternative treatments such as herbal medicines [5].

*Banha-sasim-tang* (BST, *Banxia-xiexin-tang* in traditional Chinese medicine and *Hange-shashin-to* in *Kampo* medicine) is an herbal formula containing the following seven herbs, *Pinelliae* tuber, *Scutellariae radix*, *Zingiberis rhizoma*, *Ginseng radix*, *Glycyrrhizae radix*, *Zizyphi fructus*, and *Coptidis rhizoma*. It is used for the treatment of epigastric stuffiness which, in Korean medicine, is a disease state characterized by discomfort in the epigastric region [7]. The efficacy of BST for improving GI function and its mechanism of action have been elucidated in several studies [8–10]. In the light of the findings of these studies, the present proposed randomized, double-blind, placebo-controlled, clinical trial aims to investigate the efficacy of BST for the treatment of FD.

To date, two randomized controlled trials (RCTs) of the efficacy of BST on FD have been performed. The first study evaluated the efficacy of BST in patients with FD and cold-heat complexity pattern, whereas the other included patients with common FD. The first study indicated a significant improvement occurred in the treatment group over the placebo group, whereas the other study did not indicate a significant difference in the primary outcome between the treatment and the placebo groups [11, 12]. The discrepancy in these results was likely due to pattern features specific to a type of oriental medicine (traditional Chinese, *Kampo*, or Korean medicine practices).

Oriental medicine has, for 23 centuries, considered the human body to be a holistic system, and purports that the maintenance of health occurs via a balance of this system through a unity of opposites, mutual control, and the inter-transformation of substances and functions in the body [13, 14]. An imbalance in this dynamic equilibrium causes illness. Thus, the main principle of treatment is to restore balance and correct the functional manifestation of the imbalance, which is a key factor for diagnosis in oriental medicine. Different functional manifestations and signs, which are diverse owing to each patient’s constitution and individual state, are categorized under “patterns” (called “*Zeung*” in Chinese). Patterns have been used as diagnostic tools, and for determining acupoints and medicinal herbs for treatment in oriental medicine [13, 15]. Today, patterns are considered for designing clinical trials using medicinal herbs and acupuncture.

In the above theory, the patients with FD may have been classified into multiple “patterns” and, therefore, may have required pattern classification for treatment to be effective overall. Therefore, we will perform a clinical trial using BST specifically for the treatment of excess pattern, the accumulation or stagnation of metabolic waste, body fluids, and blood.

## Methods

### Study design

**Fig. 1:**
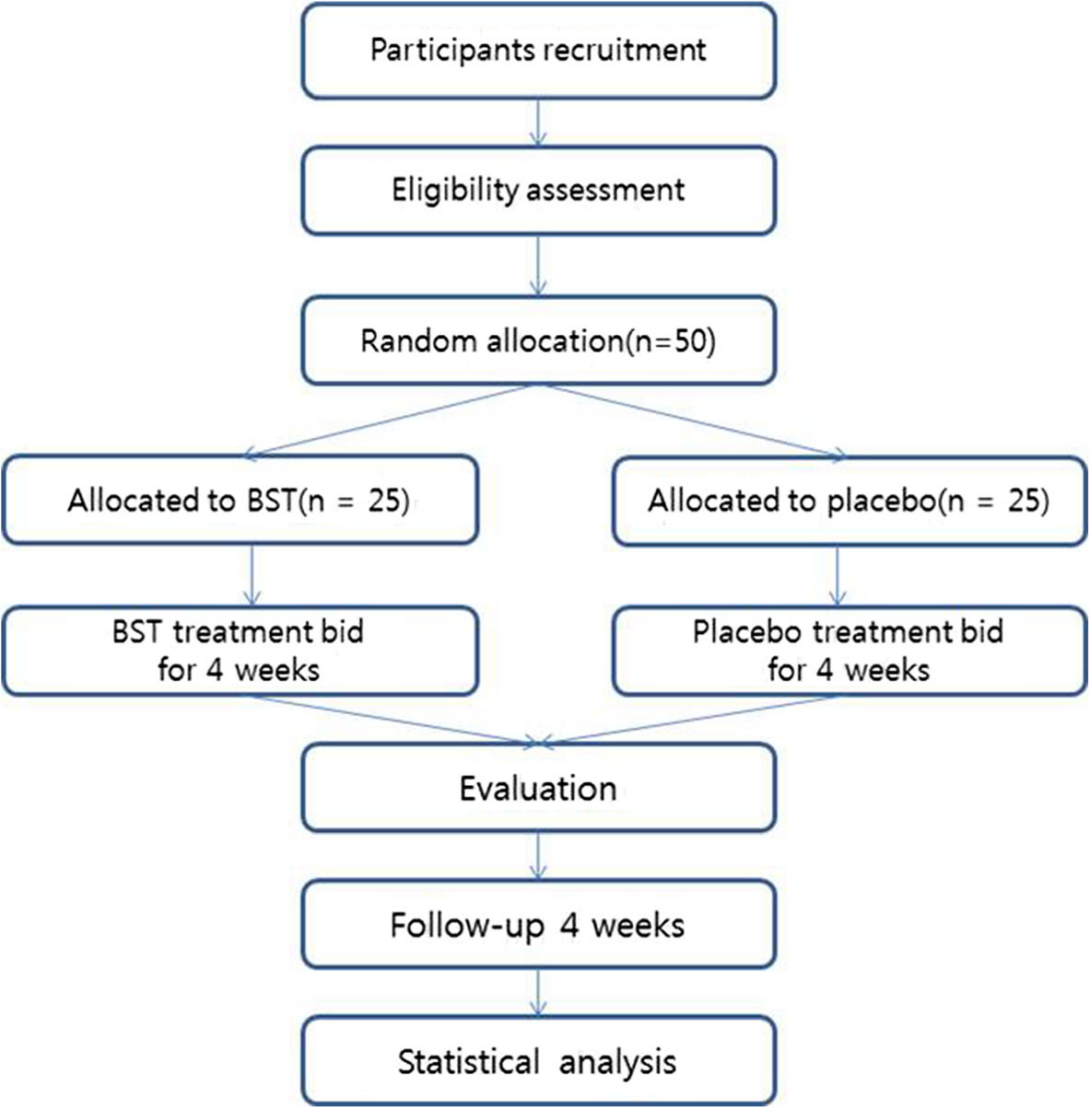
Flow chart of trial design and schedule

This trial is an investigator-initiated, randomized, blinded, parallel-group trial, in the Daejeon Oriental Hospital of Daejeon University in Daejeon, Korea, of BST versus placebo for patients with FD and excess pattern. This clinical trial will consist of 4 weeks of oral administration of BST or placebo and a 4-week follow-up period (Fig. 1). A Standard Protocol Items: Recommendations for Interventional Trials (SPIRIT) Checklist [16] is provided in Additional file 1.

### Recruitment

Participants will be recruited through advertisements in our hospital and in local newspapers.

### Population

#### Inclusion criteria

The patient inclusion criteria are as follows:

- Age 19 to 75 years with persistent dyspepsia for over 3 years and an onset of symptoms of at least 6 months prior to study enrollment
- Normal endoscopy within the previous 3 years
- Meet the ROME III criteria [17]:
  Persistent dyspepsia for over 3 months and one or more of the following symptoms: postprandial fullness, early satiety, epigastric pain or burning, and no evidence of structural disease (including in upper endoscopy) that likely explains the symptoms
- Two or more of the following eight moderate symptoms: epigastric pain, discomfort, burning, stuffiness, and fullness, as well as postprandial fullness, nausea, and early satiety; and a total of at least 6 points in the scoring of the eight symptoms using the following severity scale: 0; mild: 1; moderate: 2; and severe: 3
- Referred with an excess pattern determined using an instrument of pattern identification for FD [18]
- Ability to provide fully informed consent to participate in the study

#### Exclusion criteria

The patient exclusion criteria are as follows:

- History of GI surgery (except appendectomy)
- Presence of GI bleeding, intestinal obstruction, or GI perforation
- Confirmed or likely (current or within the previous 2 years) colorectal cancer, duodenitis or stomach cancer
- Severe hepatic dysfunction, congestive heart failure, or renal failure
- Liver enzymes, AST or ALT, increased by two or more times over the normal range
- Current or past use of non-steroidal anti-inflammatory drugs (NSAIDs) or corticosteroids
- Use of other investigational study drugs within 30 days before study entry
- Women who are pregnant or lactating. Woman of child-bearing age must be using adequate contraception
- Inability to complete relevant questionnaires

### Withdrawal, dropout, and discontinuation

Participants can voluntarily withdraw at any time during the trial, and the investigator can terminate the observation and exclude participant data if necessary. Reasons for withdrawal will be recorded in Case Report Forms (CRFs), and the last data recorded for these participants will be included in the data analysis. The trial can be terminated under the following circumstances: (1) occurrence of serious adverse events (AEs) related to the research medication, (2) at the request of the participants, (3) non-compliance to trial medication, and (4) the request for other medications to relieve the symptoms of FD.

### Intervention

Eligible patients will be asked to provide informed consent to participate in the study. Next, enrolled patients will be randomized to receive either BST or the placebo, which will both be orally administered at a dose of 10 g twice daily for 4 weeks for a total daily dose of 20 g.

#### Banha-sasim-tang (BST)

Each 10 g of the BST syrup prepared by Jeong-woo Pharmaceutical Company Ltd., (Seoul, Korea) will be produced according to Korean Good Manufacturing Practice. BST soft extract is composed of the following herbs: *Pinelliae* tuber (1.178 g), *Scutellariae radix* (0.840 g), *Ginseng radix* (0.803 g), *Glycyrrhizae radix* (0.732 g), *Zingiberis rhizoma* (0.500 g), *Zingiberis rhizoma recens* (0.077 g), *Coptidis rhizoma* (0.133 g), and *Zizyphi fructus* (0.512 g). β-Cyclodextrin (1.500 g), apple concentrate (1.350 g), carboxymethyl cellulose (50 mg), sodium benzoate (5.4 mg), and water will be included as excipients in each 10 g of BST soft extract.

#### Placebo

The placebo, which will be provided by the same supplier (Jeong-woo Pharmaceutical Company Ltd., Seoul, Korea), will contain starch (0.600 g, lactose mixture (1.412 g), caramel food coloring, and excipients and will have a color and taste similar to those of the BST soft extract with no active components. At the end of the study, participants will indicate whether they thought that the drug they were administered was the active drug or the placebo to evaluate the success of blinding.

#### Concomitant medication

Medications that may affect the efficacy of the trial medication, including NSAIDs, corticosteroids, and GI motility-related drugs will be prohibited. Patients will receive treatment for severe symptoms immediately they occur. However, the patients can be excluded under the request for other medications to relieve the symptoms of FD.

### Outcome measures

#### Primary outcome measure

The primary efficacy parameter is the change from baseline in the total score of the Nepean Dyspepsia Index (NDI) symptom-based questionnaire [19]. The NDI was developed to measure the severity of dyspepsia symptoms and is composed of two parts: a symptom-based questionnaire and a quality-of-life (QoL) evaluation. The Korean version of the NDI (NDI-K) symptom-based questionnaire, which was translated into Korean and validated [20], will be used as the primary outcome measure. The NDI-K symptom-based questionnaire scale consists of the questions about the following 15 dyspeptic symptoms: burping/belching; fullness after eating or slow digestion; nausea; inability to finish a regular meal; bad breath; regurgitation of bitter/sour tasting fluid into the mouth or throat; vomiting; and discomfort, cramps, burning sensation, pain or ache, pressure, and bloating in the upper abdomen; as well as a burning sensation (heartburn), and pain in the chest.

Three aspects, consisting of symptom severity and frequency and symptoms experienced out of the 15 items, will be assessed using a 5-point Likert scale: none, 0; slight, 1; moderate, 2; severe, 3; and very severe, 4. The NDI scale is easy for participants to understand and adequately reflects their symptoms. The NDI-K symptom-based questionnaire scale will be assessed at baseline, and week 2 and 4 during oral administration of BST, and at 4 weeks after completion of BST administration.

#### Secondary outcome measures

Outcome measures will consist of the following items:

- The efficacy parameter is a change from baseline in the total score of the QoL section of the NDI-K
- The efficacy parameter is a change from baseline in the total score of the validated FD-related QoL (FD-QoL) scale [21]
- Assessment of gastric myoelectrical dysrhythmias (GMA)
- The efficacy parameter is a change from baseline in the total score of the Visual Analog Scale (VAS) for measuring severity or pain

### Safety assessment

The safety assessment, consisting of vital sign measurements, electrocardiography, and liver and renal function tests will be conducted at visits 2 and 4.

### Randomization

In this study, patient randomization will be performed with a group allocation ratio of 1:1. The random sequence will be generated using a computer-generated random number table, and random numbers will be assigned using sealed, opaque envelopes. Both the patients and clinicians will be blinded until the end of the trial. The research team involved in the treatment administration and data collection will not have a role in the assignment process.

### Sample size calculation

The objective of this study is to validate the effect of BST treatment on FD. In this study, the sample size will be estimated based on the results of several clinical experiences and two previous studies [22, 23]. The average variation (*δ*) is assumed to be 20 and the standard deviation (SD = *σ*) is assumed to be 22.4 between the treatment and the placebo (control) groups. In addition, the patients will be allocated at a 1:1 ratio to each group and assessed a two-sided test after considering 5% significance and 80% power levels. Therefore, this study will include 40 participants, who will be divided into two groups of 20 (*n*) each:

$$ n=\frac{2\times {\left({Z}_{\alpha /2}+{Z}_{\beta}\right)}^2\times {\sigma}^2}{\delta^2}. $$

Considering a potential dropout rate of 20%, this study will require at least 50 participants.

### Monitoring

The study monitoring will follow Good Clinical Practice principles and will be processed by Korea Institute of Oriental Medicine. A clinical research associate (CRA) will be in attendance every 4 weeks to monitor and ensure the quality of the recorded data. The CRA will check the medical records, informed consent forms, source documents, and the electronic CRFs.

### Evaluation of adverse events (AEs)

AEs are any adverse, unintended changes in vital signs, symptoms, or laboratory tests that occur after patients have participated in the clinical trial. All participants will be requested to report any AEs that occur during the trial, and each AE must be recorded in the CRFs by the investigator. In addition, all AEs will be assessed for causality, and when serious AEs occur, the Institutional Review Board (IRB) and principal investigator will be notified as soon as possible. After receiving a serious AE report, the site investigator will decide whether the participant should be withdrawn from the trial.

### Data collection and analysis

This trial will last for 9 weeks, including 4 weeks of study drug administration and the follow-up period. Participants will attend four assessment visits after screening and complete a set of questionnaires and other evaluations (Table 1). Data will be collected using electronic spreadsheets, which will be completed by practice nurses.

**Table 1:**
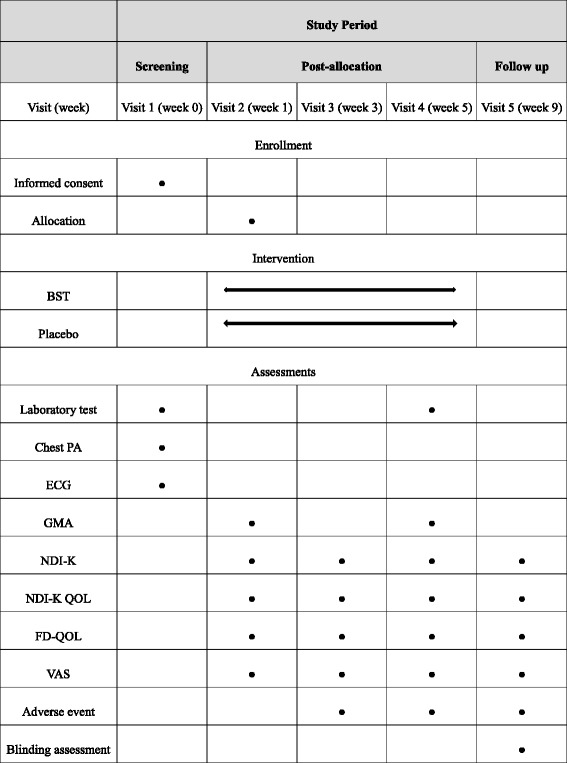
Schedule for enrollment, intervention, and assessment

*BST Banha-sasim-tang*, *ECG* electrocardiogram, *GMA* Gastric myoelectrical dysrhythmias, *FD-QoL* Functional dyspepsia-related quality of life, *NDI-K* Korean version of the Nepean Dyspepsia Index, *QoL* quality of life, *PA* posterior-anterior, *VAS* Visual Analog Scale

Data will be analyzed using the intention-to-treat analysis and will include all participants who will undergo randomization. The analysis will be performed considering each individual as a unit of analysis. All reported *p* values will be two-tailed and for each analysis, *p* < 0.05 will be considered statistically significant. Data will be described as the mean, standard deviation (SD) or frequency, percentage, and confidence interval (CI). Category-type data will be analyzed using the Pearson’s χ^2^ test or Fisher’s exact test. Measurement and rating data will be described using the Student’s *t* test and the Wilcoxon rank sum test, respectively. Missing data will be imputed using multiple imputations and the statistical analysis will be performed using the statistical analysis SAS software (version 9.4, SAS Institute Inc., Cary, NC, USA).

## Discussion

FD is not a life-threatening condition, but its symptoms interrupt daily activity [24]. FD has major socioeconomic consequences, and its yearly cost was US$18 billion in the USA in 2009 [25]. Therefore, physicians consider the investigation, diagnostic testing, and effective treatment of FD as critical to minimize potential socioeconomic consequences. Although various treatments, including acid-suppression therapy, prokinetic agents, antidepressants, *Helicobacter pylori* eradication therapy, and psychological therapy, are administered for FD, 50% of patients seek alternative options because of treatment failure [5]. Therefore, numerous patients continue to search for effective treatments to relieve FD and some use herbal medicines, such as STW5, which consists of nine herbs [5]. To evaluate the efficacy of such herbal medicines, clinical trials using decoctions or combination herbal therapies have been performed [22, 26–29]. This study will be performed to determine the efficacy of BST in FD.

The indication for BST in ancient herbal prescriptions is “epigastric stiffness” (心下痞), which is similar to early satiety and abdominal discomfort [12]. Currently, BST is used for the treatment of various GI tract diseases and symptoms, such as chronic gastritis [30] and reflux esophagitis [31]. The effects of BST may occur owing to its control of the levels of GI hormones involved in gastric motility, and the hypothalamic-pituitary adrenal axis [9, 10, 32]. Moreover, BST has been used for the treatment of diarrhea and hypogastric function in patients with cancer following chemotherapy [33–35]. As stated above, BST can be applied to several diseases with abdominal discomfort because multiple diseases can share a similar “pattern,” and thus can be cured using the same herbal remedy according to the theory of oriental medicine [15]. In this study, we will apply BST to FD patients and use “pattern” (“*Zheng*” in Chinese, translated to “syndrome”) as part of the participant inclusion criteria. This study will be limited to participants who are classified as having an excessive pattern using an instrument of pattern identification for FD in order to determine the efficacy of BST for this specific subset of patients.

In addition, we will use the NDI-K as the primary outcome for evaluating the severity of FD. The NDI-K was developed for measuring both symptoms and impairments on the dyspepsia-specific health-related QoL (H-QoL). It has proven crucial for the diagnosis of FD [19]. The NDI-K has been validated via clinical trials, and is used as an outcome assessment in many FD clinical trials [36–38]. In this study, we will evaluate the improvement of FD by assessing physical symptoms, and also the QoL using the NDI-K in patients with FD identified as having an “excessive pattern.”

### Trial status

This trial is ongoing at the time of manuscript submission.

## Additional file

Additional file 1: Standard Protocol Items: Recommendations for Interventional Trials (SPIRIT) 2013 Checklist. (DOC 124 kb)

## Abbreviations

AEs: Adverse events
BST: *Banha-sasim-tang*
FD: Functional dyspepsia
FD-QoL: Functional dyspepsia-related quality of life
GMA: Gastric myoelectrical dysrhythmias
NDI: Nepean Dyspepsia Index
NDI-K: Korean version of the Nepean Dyspepsia Index
NSAID: Non-steroidal anti-inflammatory drug
RCT: Randomized controlled trial
VAS: Visual Analog Scale
